# A clinicopathologic study of malignancy in VCP-associated multisystem proteinopathy

**DOI:** 10.1186/s13023-022-02403-9

**Published:** 2022-07-15

**Authors:** Alyaa Shmara, Mari Perez-Rosendahl, Kady Murphy, Ashley Kwon, Charles Smith, Virginia Kimonis

**Affiliations:** 1 Division of Genetic and Genomic Medicine, Department of Pediatrics, University of California-Irvine, Lab and FEDEX: Hewitt Hall, Rm 2038, Health Sciences Rd., Irvine, CA 92697 USA; 2 Department of Pathology, University of California-Irvine, Irvine, CA USA; 3 Department of Neurology and Sanders-Brown Center On Aging, University of Kentucky, Lexington, KY USA

**Keywords:** Cancer, VCP, Myopathy, Paget disease of bone, Frontotemporal dementia, IBMPFD, Multisystem proteinopathy, Peripheral nerve sheath tumor, Anaplastic pleomorphic xanthoastrocytoma, Thymoma

## Abstract

**Background:**

Valosin containing protein (VCP) is an important protein with many vital functions mostly related to the ubiquitin–proteasome system that provides protein quality control. VCP-associated inclusion body myopathy with Paget disease of bone and frontotemporal dementia, also termed VCP disease and multisystem proteinopathy (MSP 1), is an autosomal dominant disorder caused by monoallelic variants in the *VCP* gene on human chromosome 9. VCP has also been strongly involved in cancer, with over-activity of VCP found in several cancers such as prostate, pancreatic, endometrial, esophageal cancers and osteosarcoma. Since MSP1 is caused by gain of function variants in the *VCP* gene, we hypothesized our patients would show increased risk for developing malignancies. We describe cases of 3 rare malignancies and 4 common cancers from a retrospective dataset.

**Results:**

Upon surveying 106 families with confirmed *VCP* variants, we found a higher rate of rare tumors including malignant peripheral nerve sheath tumor, anaplastic pleomorphic xanthoastrocytoma and thymoma. Some of these subjects developed cancer before displaying other classic VCP disease manifestations. We also present cases of common cancers; however, we did not find an increased rate compared to the general population. This could be related to the early mortality associated with this disease, since most patients die in their 50–60 s due to respiratory failure or cardiomyopathy which is earlier than the age at which most cancers appear.

**Conclusion:**

This is the first study that expands the phenotype of VCP disease to potentially include rare cancers and highlights the importance of further investigation of the role of *VCP* in cancer development. The results of this study in VCP disease patients suggest that patients may be at an increased risk for rare tumors. A larger study will determine if patients with VCP disease develop cancer at a higher rate than the general population. If that is the case, they should be followed up more frequently and screened for recurrence and metastasis of their cancer.

## Background

### VCP disease

Inclusion body myopathy with Paget disease of bone and frontotemporal dementia (IBMPFD), also known as VCP disease and multisystem proteinopathy (MPS I), is an autosomal dominant hereditary disorder caused by monoallelic variants in the valosin containing protein (*VCP*) gene on chromosome 9p13.3–12. The protein itself has several cellular functions, many pertaining to protein quality control and homeostasis [1]. Many of the specialized functions of VCP involve the ubiquitin–proteasome system (UPS) removing ubiquitinated substrates from cellular structures (including endoplasmic reticulum, mitochondria, endosomes, chromatin, and aggresomes) and unfolding them for degradation by the 26S proteasome [1, 2]. This function is especially evident in the endoplasmic reticulum associated degradation (ERAD) pathway. VCP is also required for autophagy through its role in autophagosome maturation which is disrupted in VCP disease [3]. The failure of proper VCP activity in the UPS and autophagy leads to protein aggregation. Other roles of VCP include those related to cell cycle progression, genomic stability, and membrane trafficking [4].

Mutations in the *VCP* gene consequently lead to protein aggregation, resulting in the many manifestations of VCP disease. Inclusion body myopathy (IBM), affecting 90% of patients, is characterized by proximal muscle weakness and atrophy involving the shoulder and hip girdle muscles with onset in the 30–40 s. Skeletal muscle histology typically shows rimmed vacuoles and cytoplasmic ubiquitin and TAR DNA-binding protein 43 (TDP-43) positive inclusions [5]. Paget disease of bone (PDB) affects 42% of patients and typically manifests in the patient’s 30 s [6]. The characteristics of PDB resulting from overactive osteoclasts include elevated serum alkaline phosphatase concentration and bone pain from involvement of the hip, spine, scapulae and skull [7]. Frontotemporal dementia (FTD) usually manifests in 32% of patients in their 50–60 s. As the name implies, FTD results from degeneration of the frontal and temporal lobes of the brain. It is characterized by cognitive deficiencies, including difficulties with comprehension, dysnomia and dyscalculia in the early stages, auditory comprehension deficits, alexia, and agraphia in later stages. It also causes changes in personality and decision making [8]. We have previously reported 231 individuals carrying 15 different VCP variants in 36 families. Myopathy, PDB and FTD was present in 90%, 42% and 30% of the patients, respectively, beginning at an average age of 43, 41, and 56 years, respectively. Approximately 9% of patients with VCP variants had a classic amyotrophic lateral sclerosis (ALS) phenotype [6].

There is currently no cure or treatment for the myopathy and FTD associated with IBMPFD. PDB however can be treated with bisphosphonates which improve the pain and quality of life. Patients with IBMPFD generally die in their 50 s or 60 s due to respiratory failure or cardiac failure related to cardiomyopathy [9]. In our previous study we reported that the mean survival for the affected individuals is 62.53 years [10].

### VCP variants

More than 60 variants in the *VCP* gene have been identified in patients, proposed to be gain of function variants, the most common variant being R155H [9]. Enhanced ATPase activity has been found in disease associated variants in *VCP* [11]. *VCP* variants are also associated with amyotrophic lateral sclerosis (ALS) [12], Parkinson's disease [13], Huntington disease [14] and Charcot-Marie-Tooth disease type 2 [15].

### VCP in cancer

It has been demonstrated that the expression level of VCP is significantly elevated in several different types of cancer, including non-small cell lung carcinomas, pancreatic endocrine neoplasms, and prostate cancer [4, 16, 17]. The overexpression of VCP has been associated with poor prognosis and increased metastasis, proving it valuable as a marker for the advancement of these cancers. The inhibition of VCP has been suggested as a treatment of metastasis in certain cancers [18]. Because gain of function variants in *VCP* are implicated in VCP disease, we hypothesize that an atypical distribution of malignancy types and a higher tendency to develop cancer may be found among our patients.

### Role of VCP in cancer

VCP has been involved in the DNA damage response through the identification of multiple chromatin-associated VCP substrates such as Ku70/80 and L3M6BTL1 for DNA double-strand breaks repair [19, 20] and CDT1 during DNA replication under normal and DNA-damaging conditions [21, 22]. A recent study has found that upon DNA damage, VCP undergoes phosphorylation at Ser^784^ in its C-terminal tail, leading to the selective increase of nuclear VCP activity with respect to chromatin-associated protein degradation. High levels of nuclear pSer^784^-VCP are associated with poor outcome among patients with breast cancer who received genotoxic therapies. Thus, VCP plays an essential role in chromatin-associated protein clearance to such a degree that it is considered a ‘genome caretaker’ [23, 24].

VCP has also been shown to degrade IκBα, which is an inhibitor of the transcription factor NFκB, which when stimulated translocates into the nucleus and activates the expression of genes that stimulates cell growth, protecting the cells from apoptosis. Overexpression of the *VCP* gene correlates with constantly activated NFκB and may indeed promote cell proliferation and cell survival [4]. The NF-kB signaling pathway plays a key role in osteoclastogenesis and is well known to be a major player in PDB [25, 26] and metastasis of osteosarcoma [27]. In vitro studies indicate that cells transfected with the A232E mutant VCP showed significantly higher clearance of IκBα and increased levels of NFκB compared to cells transfected with wild type VCP [28]. Thus, *VCP* variants cause inappropriate activation of the NF-kB signaling cascade and could contribute to the mechanism of pathogenesis in multiple tissues.

Recent evidence supports the concept that VCP acts as a regulator of cellular metabolism through its link to multiple metabolic processes in cancer cell lines and in patient-derived multiple myeloma cells. Cellular VCP dependency to maintain proteostasis was increased under conditions of glucose and glutamine limitation in a range of cancer cell lines. VCP maintains cancer cell metabolic and protein homoeostasis through its correlation with GCN2 (general control nonderepressible 2), a serine/threonine-protein amino acid-sensing kinase, that plays a key role in modulating amino acid metabolism as a response to nutrient deprivation [29].

## Methods

### Patient population and ethics

This retrospective study included individuals from a large cohort of 231 patients from 106 families with familial VCP variants. Those families were surveyed for rare as well as common cancers. The survey was performed through reviewing patients’ medical records and conducting a patients’ questionnaire. Written informed consent was obtained from all individuals. This study was approved by the University of California Irvine Institutional Review Board (IRB) (#2009–1005). The study is also listed ClinicalTrials.gov (Identifier: NCT01353430).

### Histological staining

Hematoxylin and Eosin (H&E) and immunohistochemistry (IHC) staining was performed on tissue using standard protocols [5].

## Results

The study included individuals known to have cancer from a large cohort of 231 patients from 106 families with familial VCP variants. We found uncommon tumors in patients with VCP disease causing variants, some of whom presented with cancer prior to displaying classic VCP disease manifestations. These tumors include malignant peripheral nerve sheath tumor, anaplastic pleomorphic xanthoastrocytoma and thymoma. (Table 1). We also performed a survey of cancers in our cohort and discuss the cancers, both rare and common that were also reported by the subjects.

**Table 1:** Clinical features of patients reported with VCP disease and malignancies

Case	Cancer Type	Current Age y (Age deceased)	Sex	*VCP* variant (c.DNA;protein)	Myopathy/Age of onset y	PDB/Age of onset y	FTD/Age of onset y	Cancer age of onset y	Rate in VCP disease	Incidence in general population
1	Peripheral nerve sheath tumor	56	M	c.464G > A; p.R155H	+ /36	+ /33	–	45	1/231	1/100000[30]
2	Pleomorphic xanthoastrocytoma	(35)	M	c.572G > A; p.R191Q	–	–	–	32	1/231	< 1/285714[31]
3	Thymoma	(48)	F	c.463C > T; p.R155C	+ /45	–	–	30	1/231	1/769231[32]
4	Breast invasive ductal carcinoma	76	F	c.476G > A; p.R159H	–	–	+ /71	35	1/231	1/8 [33]
5	Pancreatic ductal adenocarcinoma	(55)	M	c.463C > T; p.R155C	+ /50	+ /40	–	54	1/231	1/7576[34]
6	Endometrial adenocarcinoma	(60)	F	c.463C > T; p.R155C	+ /34	+ / 45	+ /59	55	1/231	1/6667[35]
7	Prostate adenocarcinoma	72	M	c.464G > A; p.R155H	+ /33	-	–	69	1/231	1/14[36]

*FTD* = frontotemporal dementia. *PDB* = Paget disease of bone

### Case reports

#### Case 1

**Fig. 1:**
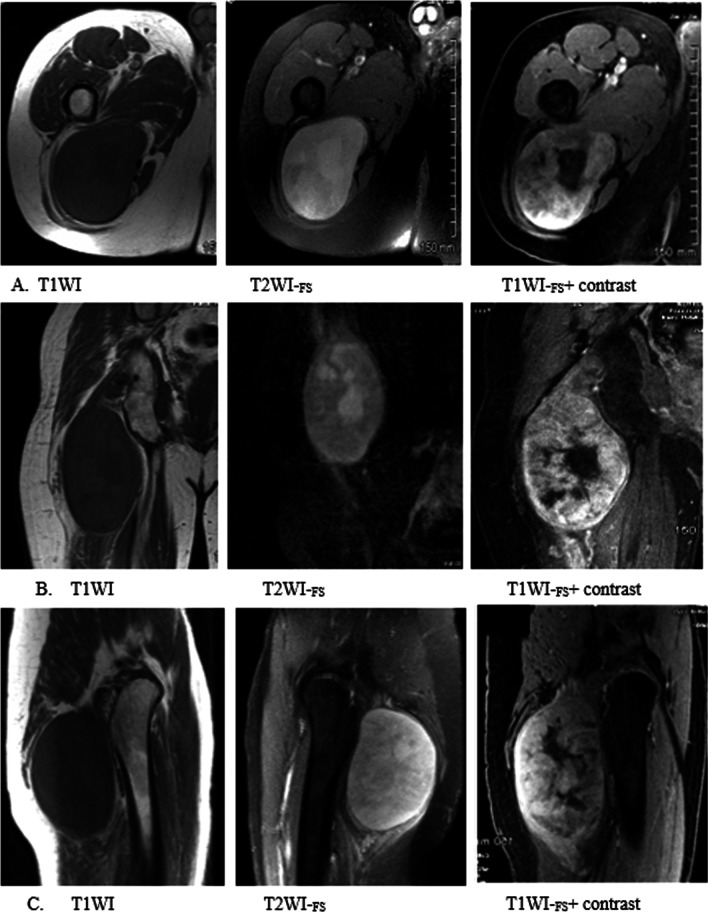
Case 1 MRI of the gluteal and pelvic area. Imaging displays a large heterogeneous enhancing mass in the posterior lateral soft tissues of the right gluteal region. Low-grade peripheral nerve sheath tumor deep to the right gluteus maximus.**A**- Axial **B**. Coronal **C**. Sagittal MRI. T1WI = T1 weighted image. T2WI-_FS_ = T2 weighted, fat-suppressed. T1WI-_FS_ + contrast = T1 weighted, fat suppressed with contrast

**Fig. 2:**
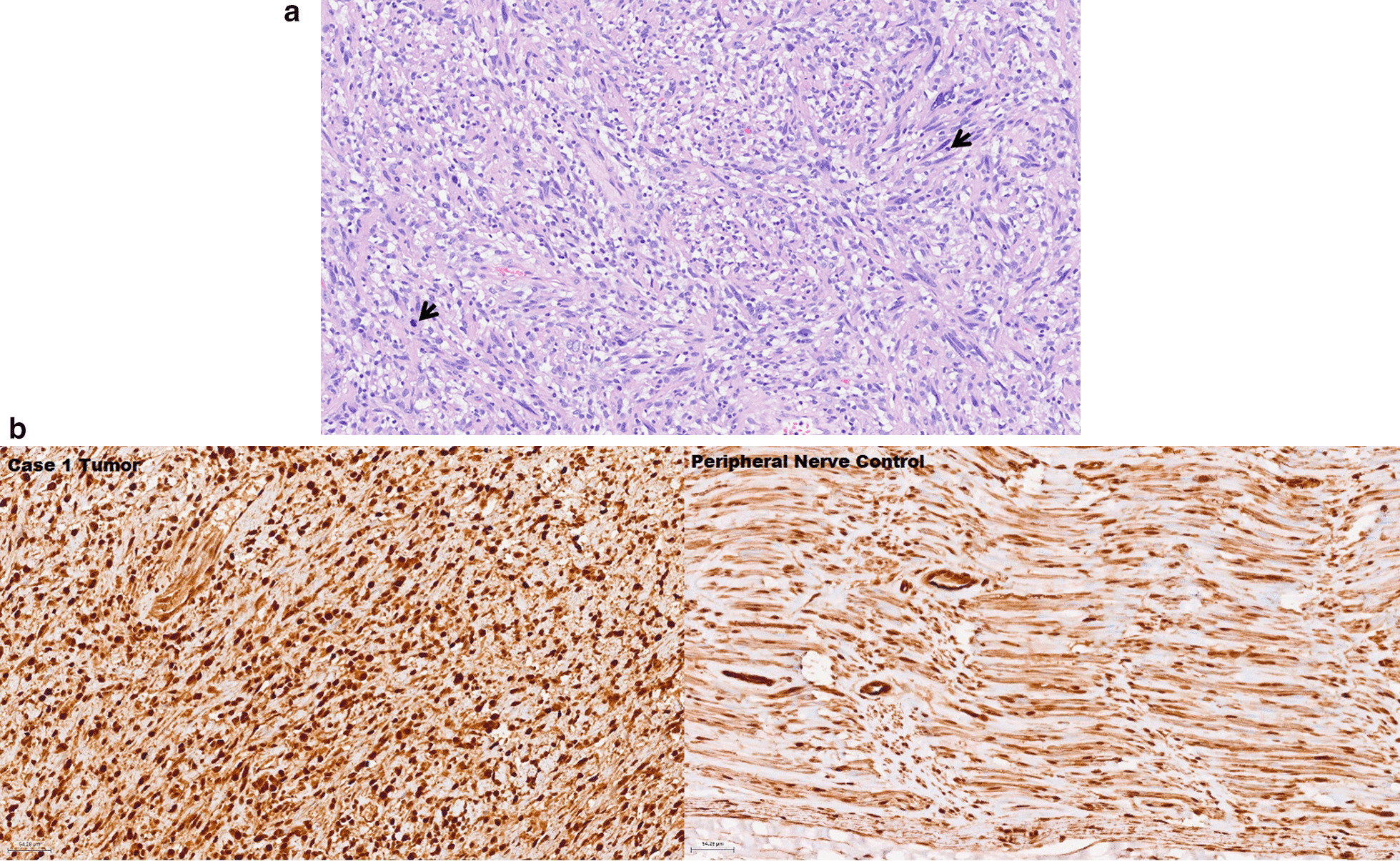
**a**. Histologic features of tumor resection from right gluteal region of Case 1. Section showing low-grade malignant peripheral nerve sheath tumor with hypercellular fascicles of mitotically active (arrows) spindle cells with enlarged hyperchromatic nuclei. (H&E, 200x) **b**. Immunohistochemistry of case 1 tumor and peripheral nerve control. The staining showed strong nuclear and cytoplasmic reactivity with mild differences between the control and the tumor

A 45-year-old previously healthy male presented with right gluteal pain and was found to have a firm and painful swelling inferior to the gluteal muscle. An MRI scan of the gluteal and pelvic area showed a 9.7 × 7.3 × 18 cm heterogeneously enhancing mass, deep to the right gluteus maximus in close proximity to the sciatic nerve (Fig. 1). Needle biopsy revealed a neurofibroma with classic histology including spindle cells admixed with shredded carrot-like collagen in a myxoid stroma. Although the biopsy did not show features of malignancy, transformation to malignant peripheral nerve sheath tumor was suspected given the imaging features and very large size. Tumor resection contained areas of neurofibroma and revealed large areas of fascicular growth pattern with marked hypercellularity, mitotic activity (up to 4 mitoses in 10 high power fields) and Ki67 proliferation index of 15%. These findings confirmed transformation to a low grade malignant peripheral nerve sheath tumor (Fig. 2a). We performed IHC staining on the tumor to look for increased VCP expression in comparison to normal peripheral nerve tissue. The staining showed strong nuclear and cytoplasmic reactivity with mild differences between the control and the tumor (Fig. 2b). The patient responded well to post-operative adjuvant 3D conformal radiation therapy.

At age 47 years, he experienced right sided foot drop, numbness of the right leg with right calf atrophy which was attributed to right sciatic neuropathy occurring after tumor resection. Imaging showed no recurrent or residual tumor; however, EMG revealed widespread denervation in both lower extremities, indicating a more global neurological condition. Several of his siblings, his father, and other paternal relatives known to have the c.464G > A, p.R155H variant in the *VCP* gene did not have other malignancies [37, 38]. Family history, however, revealed that his maternal grandfather had laryngeal cancer.

#### Case 2

**Fig. 3:**
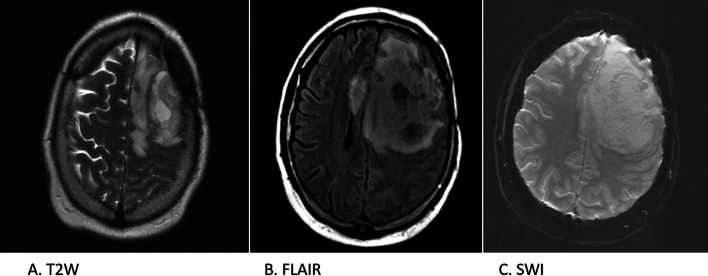
Case 2 brain MRI axial plane. Imaging shows lobulated heterogeneous solid left posterior frontal mass with foci of internal hemorrhage and surrounding edema causing significant mass effect and mild to moderate midline shift. **A**. T2WI = T2 weighted image. B. FLAIR = Fluid attenuated inversion recovery. **C**. SWI = Susceptibility weighted imaging

**Fig. 4:**
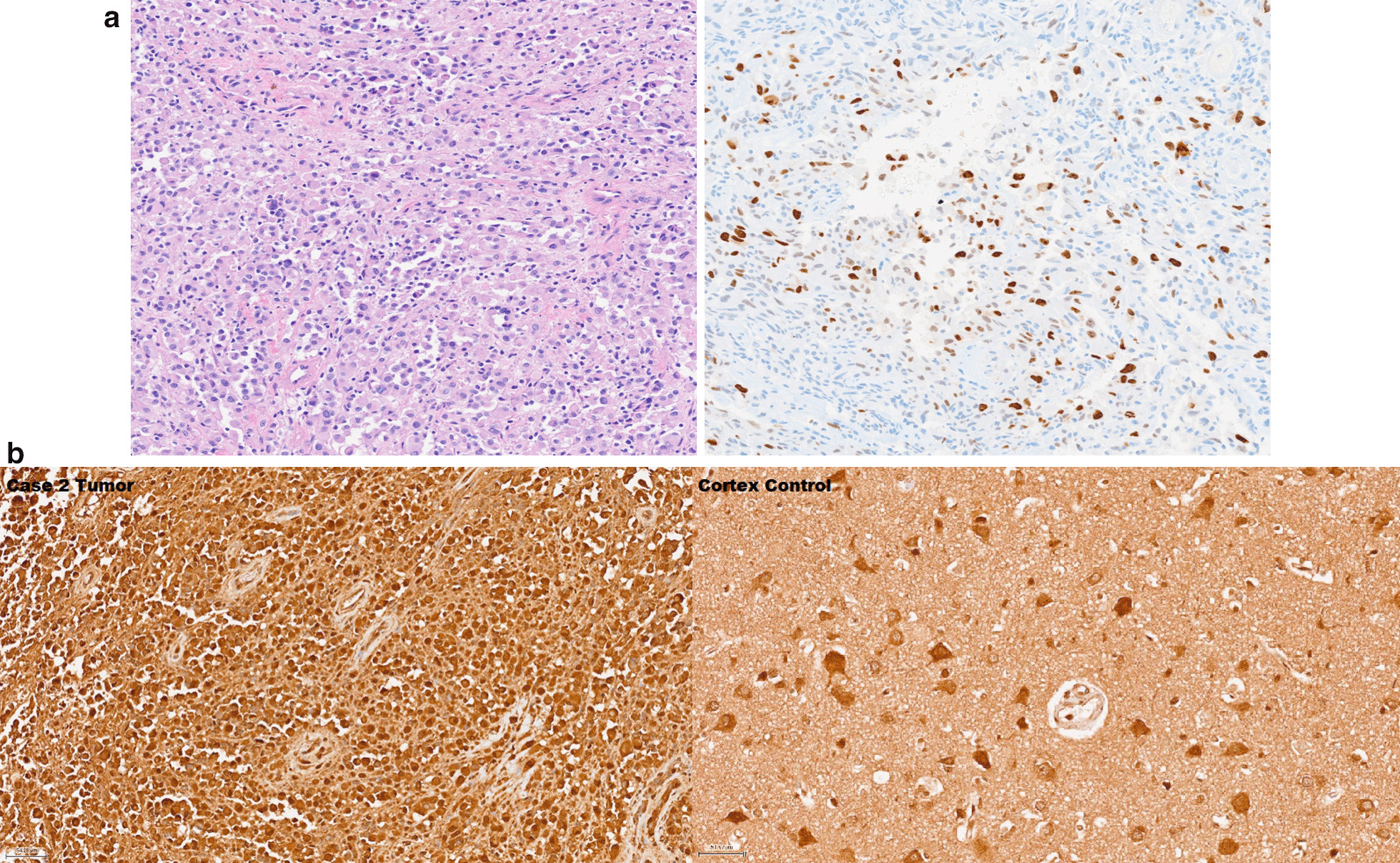
**a** Histologic features of tumor resection from left posterior frontal lobe of Case 2. Anaplastic pleomorphic xanthoastrocytoma composed of epithelioid glial cells with minimal pleomorphism (**A**) (H&E, 200x). Anaplastic features include less pleomorphism than is typical for low grade PXA, areas of necrosis (not pictured), and increased proliferation evidenced by Ki67 (**B**) (Immunohistochemistry for Ki67, 200x). **b**. Immunohistochemistry of case 2 tumor and frontal cortex control. The staining shows strong nuclear and cytoplasmic reactivity with mild increase in the staining in patient tumor cells

A 32-year-old previously healthy male underwent a brain MRI for a research study which incidentally found an enhancing lesion in the left frontal lobe. MRI findings showed a 3.4 cm lobulated left posterior frontal mass with heterogeneous enhancement and surrounding edema causing a 2 mm midline shift (Fig. 3). Subsequently, he suffered a seizure and was treated with levetiracetam. A month later the patient underwent surgical resection which showed a glial neoplasm with pleomorphic epithelioid and spindled tumor cells, eosinophilic granular bodies, and foci of lymphoplasmacytic inflammation which are consistent with a pleomorphic xanthoastrocytoma (PXA). Anaplastic features were noted, including necrosis and an elevated Ki67 proliferation index (Fig. 4a). We performed IHC staining on the tumor and compared VCP expression in the tumor versus normal frontal cortex tissue. The staining shows strong nuclear and cytoplasmic reactivity with mild increase in the staining in patient tumor cells. (Fig. 4b).

A postoperative MRI one month later showed post-surgical changes in the left frontal lobe with enhancement at the periphery of the surgical bed, which was favored to represent surgical changes and not residual tumor. After surgery he had no residual neurologic deficits and underwent radiation therapy receiving 1.8 Gy to 59.4 Gy.

A brain MRI performed 5 months later revealed hemorrhage with residual nodular thickening along the periphery of the post-surgical bed. A year later, neuroimaging showed further tumor progression with an increased amount of enhancing disease and FLAIR (fluid attenuated inversion recovery) changes. A second resection showed recurrent anaplastic PXA. Unfortunately, his post-operative MRI two months later showed return of nodular enhancement in the inner posterior superior aspect of the resection cavity. Subsequent scans showed further significant tumor progression.

He had two further resections and was treated with temozolomide followed by a combination of carmustine and bevacizumab chemotherapy for his progressive anaplastic PXA. Molecular testing of the tumor was positive for *BRAF* p.V600E (c.1799 T > A) variant. His treatment was changed to vemurafenib, a BRAF inhibitor along with bevacizumab [39].

The patient had another seizure and began suffering from dysfluent speech with difficulty naming objects. Over the next month, the patient’s aphasia worsened, and he suffered from additional breakthrough seizures and memory loss. Follow-up MRIs revealed marked increase in contrast enhancement that extended to involve the corpus callosum and the basal ganglia. His tumor grew 50% over the course of 5 weeks and was deemed inoperable. Radiation was not an option given prior heavy radiation treatment. The patient passed away at 35 years of age.

The patient was a known presymptomatic carrier of the c.572 G > A, p.R191Q variant of the *VCP* gene [10] and did not manifest prior myopathy or Paget disease. However, the aphasia could potentially have been exacerbated by the *VCP* variant. On review of his family history, the patient's father, grandfather, and great uncle passed away from myopathy and dementia between the ages of 67 and 69 years of age. Family history was not significant for cancer.

#### Case 3

This female patient was diagnosed with thymoma with metastasis to both kidneys and lungs when she was 30 years old. She was treated with surgical excision and responded well to chemotherapy post-operatively. Although her history of thymoma put her at an increased risk of myasthenia gravis [40], a test for muscle-specific receptor tyrosine kinase antibodies and for acetylcholine receptor binding antibodies performed later at age 46 years was negative.

The onset of her symptoms began at age 45 years with lower back muscle weakness progressing to weakness of the leg muscles. At age 48 years, she was wheelchair-bound with quadriparesis and had severe dysarthria, which was diagnosed as generalized ALS. She progressed very rapidly and died three months after her diagnosis of ALS. Autopsy revealed extensive spinal motor neuron degeneration with loss of spinal anterior horn cells, gliosis and mislocalization of TDP-43 positive inclusions and Bunina bodies in the hypoglossal nucleus [41].

She had a known family history of VCP disease and was a known presymptomatic heterozygote carrier for the c.463C > T, p.R155C variant. On review of her family history, her father had myopathy and dementia, her brother and sister and daughter had progressive myopathy [42]. There is no family history of cancer.

#### Case 4

**Fig. 5:**
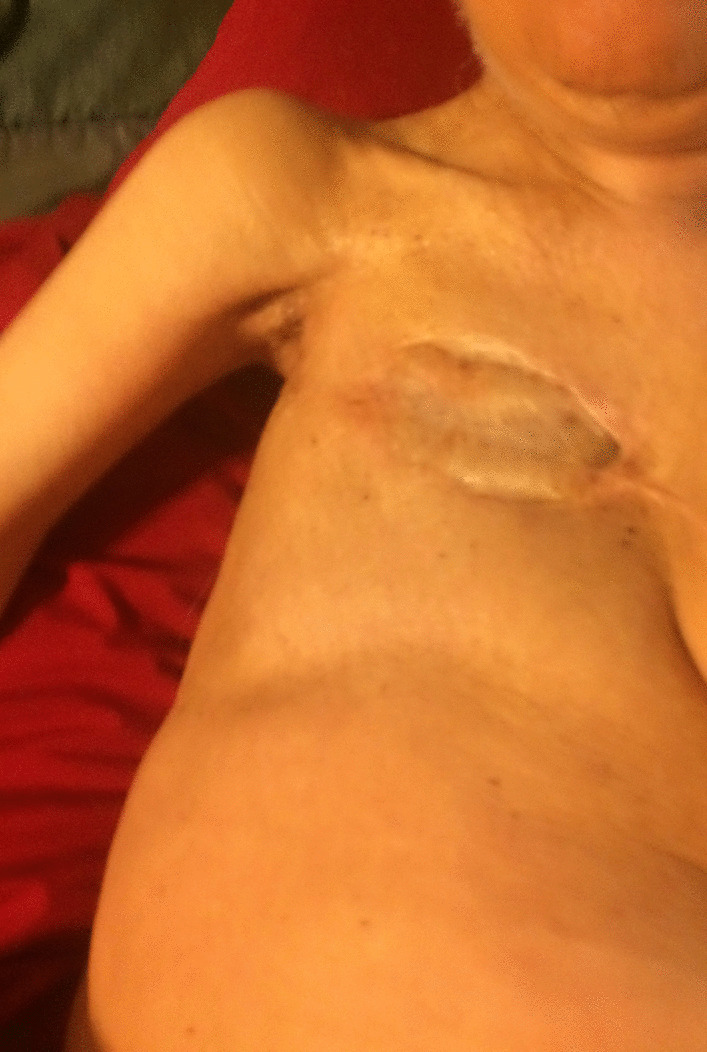
Chest of Case 4 at age 76 years. Chest photograph 41 years after radical mastectomy shows severe atrophy of pectoralis major muscle dating from irradiation in childhood

A 76-year-old female was diagnosed with an invasive ductal carcinoma of the breast at age 35 years. Since the tumor involved her entire right breast, she was treated with radical mastectomy, followed by chemotherapy and radiotherapy (Fig. 5). Surgery revealed that tumor invasion was into the deep dermis with perineural infiltration. There was no axillary lymphatic tissue noted which was attributable to prior intense orthovoltage radiation at age one year following excision of a right axillary mass for which no medical records were available.

At the age of 71 years, the patient suffered from deteriorated behavioral and mental condition and was suspected to have Alzheimer’s disease. She was later diagnosed with FTD associated with the *VCP* c.476G > A, p.R159H variant previously reported in an Austrian family with VCP disease [43]. Her family history was significant for a brother who was diagnosed with IBM attributed to the same *VCP* variant. In addition, the patient’s mother, aunt and older sister had already passed from dementia assumed to be FTD associated with VCP disease. There was no reported family history of cancer.

#### Case 5

A male patient was diagnosed with myopathy, PDB and type 2 diabetes mellitus at the age of 50 years and developed pancreatic cancer 4 years later. The patient’s CT scan showed a heterogeneous 5.3 cm. × 3.2 cm. mass in the head and uncinate process of the pancreas that surrounded the superior mesenteric vein and invaded the portal vein, and also lesions in his liver and spleen. Biopsy of the pancreatic mass showed well-differentiated ductal adenocarcinoma. The patient was known to have the c.463C > T, p. R155C *VCP* variant. The patient’s family history was significant for a brother with PDB, a father with myopathy, PDB and dementia, and several other family members with familial myopathy [42]. His sister, case 6 was diagnosed with endometrial cancer.

#### Case 6

A female patient with the c.463C > T, p. R155C variant developed distal myopathy at the age of 34 years which initially only affected her hands. This progressed to overall muscle weakness that resulted in frequent falls and trouble ambulating. By age 58 years, the patient was wheelchair-bound and had a Foley catheter.

She was diagnosed with endometrial adenocarcinoma at age 55 years after several months of experiencing postmenopausal bleeding. Endometrial biopsy showed invasive grade 2 endometrioid adenocarcinoma with myometrial invasion for which she underwent hysterectomy and received chemo and radiation therapy post-operatively. She died at age 60 years from complications of her underlying myopathy and dementia.

#### Case 7

A 72-year-old male first noted distal and proximal muscle weakness in his twenties. Muscle biopsy revealed rimmed vacuoles characteristic of IBM. There was no evidence of PDB or dementia. This patient was diagnosed with aggressive prostatic adenocarcinoma with metastatic adenopathy at age 69 years discovered by CT scan. The patient was not eligible for chemotherapy, radiotherapy, or surgery due to his debilitated state. Instead, he was started on androgen deprivation therapy with leuprolide and bicalutamide. Consequently, leuprolide was stopped due to the development of nausea and weakness, and treatment was continued with bicalutamide, which controlled tumor progression. The patient had the c.464G > A, p.R155H variant and displayed features of IBM as do several maternal family members who have been previously reported [42, 44]. Family history of cancer was significant for the patient’s father and paternal grandmother having renal cancer.

## Discussion

### Hypothesis for increased risk for cancer in VCP disease

It was hypothesized that VCP variants in patients may play a role in up-regulating the expression or activity of VCP, leading to an increased rate of tumors in these patients. We indeed found that several patients were found to have uncommon tumors.

Peripheral nerve sheath tumors are usually benign tumors that arise in peripheral nerves or extraneural soft tissue. Malignant peripheral nerve sheath tumors (MPNST) are uncommon and aggressive tumors that usually present in young or middle-aged adults and can either arise from a pre-existing neurofibroma or occur de novo. MPNST has a reported incidence of 0.001% in the general population [30]. In VCP disease however, the rate is 0.433%. About 50% of MPNST are associated with an autosomal dominant variant in the neurofibromin 1 (*NF1*) gene that causes multiple neurofibromas: this variant leads to a loss of function in the tumor suppressor neurofibromin [45]. However, up to 47% of MPNSTs develop sporadically [46]. Case 1 did not have any clinical features of NF1 apart from the tumor itself. This suggests that other pathways are implicated in neurofibroma transformation to aggressive MPNST. EGFR-STAT3 signaling pathway was found to promote such transformation to aggressive MPNST [30]. Interestingly, VCP promotes the growth, invasion, and metastasis of colorectal cancer through activation of STAT3 signaling [47].

Pleomorphic xanthoastrocytoma (PXA) is a rare type of brain cancer which most commonly affects children or young adults [48]. It is difficult to estimate the incidence of PXA in the general population since the incidence of anaplastic astrocytoma is 3.5 per million person/year [31] yet, the rate of PXA in VCP disease is 1/231. The diagnosis of anaplasia in PXA is based upon the presence of increased mitotic activity; however, the factors that drive progression to anaplastic PXA are not well-defined. Most PXAs harbor RAF alterations (most frequently *BRAF V600E* variant) plus homozygous deletion of the *CDKN2A* tumor suppressor, and anaplastic PXAs often also have *TERT* gene alterations [49].

Overexpression of *VCP* correlates with constantly activated NFkB and may promote cell proliferation [4]. In a study by Aladhraei et al., 11 out of 13 NF-κB positive colorectal tumors were involved with positive lymph nodes metastasis and showed strong association with the high Ki67 expression [50]. In another study, NF-κB expression was significantly correlated with Ki67 expression in the metastatic lymph node tissues of colorectal cancer [51]. Interestingly, both case 1 and case 2 showed elevated Ki67 proliferation indices. This suggests that VCP variants could contribute to the mechanism of pathogenesis in multiple tissues through causing inappropriate activation of the NF-kB signaling cascade.

Thymoma, likewise, is a rare type of cancer arising in thymic epithelial cells, usually presenting in a patient’s 40 s or 50 s. The incidence of thymoma in the US is 0.13 per 100,000 person-year [32]. However, the rate of thymoma in VCP disease is 1/231. Treatment involves surgery and adjuvant radiation because of its sensitivity to chemotherapy and radiation [52]. The early age of presentation in case 3 may be caused by the patient’s *VCP* variant.

Studies of thymoma reveal missense variant in the *GTF21* gene on chromosome 7 c.74146970 T-A, with 82% prevalence in type A thymoma and 74% prevalence in type AB thymoma [53]. In thymic carcinomas, a type of cancer similar to thymoma, cancer gene variants in genes such as *TP53* and *CDKN2A* have been identified [54]. Unfortunately, we were not able to obtain tissue from this patient because the tumor occurred over 20 years ago.

We also reported other cases of cancer in our cohort of 231 patients with VCP disease in which the rate of common cancers was not higher than the general population. This might be explained by the short life span of VCP disease patients since they die from respiratory or cardiac failure [6]. Case 4 presented with aggressive breast cancer at an early age without a positive family history of breast cancer. Although breast cancer risk is increased in women treated for childhood cancer with radiation applied to a broad field of breast tissue [55], we believe that the aggressive breast tumor in case 4 was possibly exacerbated by her *VCP* variant. One study reported that VCP expression levels in breast tumors correlates with the TNM stage used to describe the amount and spread of cancer (T represents tumor size and spread into nearby tissue; N: cancer spread to nearby lymph nodes; and M: metastasis), and Ki67 proliferation marker. Compared to normal mammary epithelial cells, the expression of VCP was significantly higher in the cytoplasm of breast cancer cells. Interestingly, patients with high levels of VCP expression had poorer overall survival; therefore, VCP expression is suggested as an independent prognostic factor in breast carcinoma [56].

### Up-regulation of VCP in association with IBMPFD and with cancer

Using E-coli Rosetta cells to induce expression of disease-related *VCP* variant, a study found that all the mutants had increased ATPase activity compared to those of the wild type [11]. The A232E variant, which correlates to the most severe disease phenotype [42], showed the highest increase of activity out of all the variants tested. Thus, *VCP* variants can have a significant impact on VCP-related pathways involving ATPase activity.

It has been well-documented that VCP is overexpressed in multiple types of cancers and has been suggested as a marker for prognosis. However, it has not been shown why VCP is overexpressed in these tumors or the effects of its overexpression. Given that VCP has many different roles in the cell, including those in cell death and homeostasis, it may be challenging to identify exactly how overexpressed VCP functions differently in tumors.

Although its exact role in cancer activity remains unknown, it has been shown that VCP inhibitors can result in cancer cell death, suggesting the possible use of VCP inhibition as a potential cancer therapy [45, 57]. One potent and specific VCP inhibitor called NMS-873 was able to activate the unfolded protein response and thus interfered with autophagy, resulting in cancer cell death [57]. Another potent and specific VCP inhibitor called CB-5083, which is orally bioavailable, is responsible for causing modulation in VCP related pathways, resulting in apoptosis and antitumor activity [58].

In the case of ovarian cancer specifically, *VCP* has been found to be an essential gene in cell lineages, and a study showed that ovarian cancer was sensitive to VCP inhibition. The same study showed that modification of ER stress by agents could enhance the cytotoxic activity of VCP inhibitors in treating ovarian cancer [59].

### Rationale for the association of rare tumors in VCP disease

With experimental evidence of the up-regulation of ATPase activity in VCP disease causing variants, we propose that the *VCP* variant may be a key factor in the pathogenesis of these uncommon cancers, since *VCP* has been shown to be overexpressed in several different cancers. Targeting VCP as a potential cancer treatment has been an effective method in multiple studies [26, 53–55]. In 1990, Fearon and Vogelstein proposed a model of colorectal carcinoma resulting from variants in salient genes, including the inactivation of tumor suppressor genes and the activation of oncogenes. Accumulation of these mutations happens sequentially, with mutations of some genes preceding that of others, this process takes place over several years before the cancer develops [60]. Because patients with VCP disease have a shorter life span attributable to respiratory muscle weakness and cardiomyopathy [61], the rate of reported cancers is predicted to be lower than the general population which was the case for common cancers. Nevertheless, we found rare tumors that occurred earlier and appeared to be more aggressive in our cohort of patients with VCP disease. Since cancer is associated with overexpression of VCP, and the disease is associated with gain of function variants in the *VCP* gene, it is predicted that VCP inhibitors potentially would be effective for treatment of both disorders. Although active cancer screening for patients with germline VCP variants is not recommended, if those patients develop cancer, they need to be followed up closely for signs of local invasion or metastasis.

## Conclusion

Expression of *VCP* in cancers has been correlated with tumor aggressiveness and prognosis. However, the underlying molecular mechanism is unclear. Based on the evidence for a strong relationship between cancer and VCP function, and up-regulation of VCP activity caused by IBMPFD-related *VCP* variants, we conducted this study to characterize cancers in this group of patients. The results of this study in VCP disease patients suggests that patients may be at an increased risk for cancer, especially for rare tumors. This suggests that cancer patients with gain of function *VCP* variants may potentially be more effectively treated with VCP inhibitors. A potential topic of future research would be to further study the molecular mechanisms implicating VCP in human cancer cells.

## Data Availability

The datasets generated during and/or analyzed during the current study are available from the corresponding author on reasonable request.
